# Rotenone, Paraquat, and Parkinson’s Disease

**DOI:** 10.1289/ehp.1002839

**Published:** 2011-01-26

**Authors:** Caroline M. Tanner, Freya Kamel, G. Webster Ross, Jane A. Hoppin, Samuel M. Goldman, Monica Korell, Connie Marras, Grace S. Bhudhikanok, Meike Kasten, Anabel R. Chade, Kathleen Comyns, Marie Barber Richards, Cheryl Meng, Benjamin Priestley, Hubert H. Fernandez, Franca Cambi, David M. Umbach, Aaron Blair, Dale P. Sandler, J. William Langston

**Affiliations:** 1 The Parkinson’s Institute, Sunnyvale, California, USA; 2 Epidemiology Branch, National Institute of Environmental Health Sciences, National Institutes of Health, Department of Health and Human Services, Research Triangle Park, North Carolina, USA; 3 Veterans Affairs Pacific Islands Health Care System, Honolulu, Hawaii, USA; 4 Toronto Western Hospital, University of Toronto, Toronto, Ontario, Canada; 5 Departments of Neurology and Clinical and Molecular Neurogenetics, University of Lubeck, Lubeck, Germany; 6 Institute of Cognitive Neurology, Institute of Neuroscience, Favaloro University, Buenos Aires, Argentina; 7 Westat Inc., Durham, North Carolina, USA; 8 Center for Neurological Restoration, Cleveland Clinic, Cleveland, Ohio, USA; 9 Department of Neurology, University of Kentucky, Lexington, Kentucky, USA; 10 Biostatistics Branch, National Institute of Environmental Health Sciences, National Institutes of Health, Department of Health and Human Services, Research Triangle Park, North Carolina, USA; 11 Occupational and Environmental Epidemiology Branch, Division of Cancer Epidemiology and Genetics, National Cancer Institute, National Institutes of Health, Department of Health and Human Services, Bethesda, Maryland, USA

**Keywords:** aging, agricultural epidemiology, environmental epidemiology, epidemiology, fungicides, herbicides, insecticides, persistent organic pollutants, pesticides

## Abstract

**Background:**

Mitochondrial dysfunction and oxidative stress are pathophysiologic mechanisms implicated in experimental models and genetic forms of Parkinson’s disease (PD). Certain pesticides may affect these mechanisms, but no pesticide has been definitively associated with PD in humans.

**Objectives:**

Our goal was to determine whether pesticides that cause mitochondrial dysfunction or oxidative stress are associated with PD or clinical features of parkinsonism in humans.

**Methods:**

We assessed lifetime use of pesticides selected by mechanism in a case–control study nested in the Agricultural Health Study (AHS). PD was diagnosed by movement disorders specialists. Controls were a stratified random sample of all AHS participants frequency-matched to cases by age, sex, and state at approximately three controls: one case.

**Results:**

In 110 PD cases and 358 controls, PD was associated with use of a group of pesticides that inhibit mitochondrial complex I [odds ratio (OR) = 1.7; 95% confidence interval (CI), 1.0–2.8] including rotenone (OR = 2.5; 95% CI, 1.3–4.7) and with use of a group of pesticides that cause oxidative stress (OR = 2.0; 95% CI, 1.2–3.6), including paraquat (OR = 2.5; 95% CI, 1.4–4.7).

**Conclusions:**

PD was positively associated with two groups of pesticides defined by mechanisms implicated experimentally—those that impair mitochondrial function and those that increase oxidative stress—supporting a role for these mechanisms in PD pathophysiology.

Mitochondrial dysfunction and oxidative stress have long been implicated as pathophysiologic mechanisms underlying Parkinson’s disease (PD) (Betarbet et al. 2000; Di Monte et al. 2002). Genetic forms of PD associated with mutations in the *alpha-synuclein, PARKIN, PINK1,* or *DJ-1* genes may involve these mechanisms (Henchcliffe and Beal 2008). In experimental models, the pesticides paraquat, which causes oxidative stress, and rotenone, which inhibits mitochondrial complex I, both induce loss of nigral dopaminergic neurons and behavioral changes associated with human PD (Henchcliffe and Beal 2008). Yet despite decades of laboratory study, neither pesticide has been definitively associated with PD in humans. Previous studies have reported associations with paraquat, but results are inconsistent and, in general, studies included few exposed cases; evidence concerning rotenone is sparse (Table 1). To assess the relevance of experimental results to human PD, we investigated the association of PD with use of pesticides linked to complex I inhibition or oxidative stress in a population with well-characterized pesticide exposure.

The Farming and Movement Evaluation study (FAME) is a case–control study nested in the Agricultural Health Study (AHS), a prospective study including 84,740 private pesticide applicators (mostly farmers), recruited in 1993–1997 in Iowa and North Carolina, and their spouses (Alavanja et al. 1996). Pesticide use is reported reliably by these applicators (Blair et al. 2002; Hoppin et al. 2002). We previously reported that self-reported PD was associated with increasing lifetime days of use of any pesticide, but no specific pesticide could be definitely implicated (Kamel et al. 2007). In this study, we assessed lifelong use of pesticides with toxicant mechanisms relevant to PD. Because 15–25% of self-reported PD diagnoses in community populations may be incorrect (Tolosa et al. 2006), we based diagnoses on in-person examination and consensus of two experts, minimizing diagnostic misclassification and enabling analyses of clinical features. We report here results from our investigation of pesticides previously associated with mitochondrial complex I inhibition (termed “complex I inhibitors”) or oxidative stress (termed “oxidative stressors”).

## Methods

### Case and control identification

From among AHS cohort members who were private pesticide applicators (mostly farmers) and their spouses, we identified persons suspected to have PD based on diagnoses from self-reports or state mortality files. We selected potential controls by stratified random sampling of all AHS participants. Cohort members who were ultimately diagnosed with PD or parkinsonism, dead, cognitively impaired, or seriously ill were not eligible to be controls. Controls were frequency-matched to cases by age (< 40, 40–49, 50–59, 60–64, 65–69, ≥ 70 years), sex, and state (Iowa or North Carolina) at a ratio of approximately three controls per case.

Living suspect cases and all potential controls were assessed during home visits. Movement disorder specialist neurologists (subsequently called “neurologists”) assessed all cases and 5% of controls. Neurologist-trained technicians assessed the remaining controls. The in-person assessment included factors used to diagnose PD and to distinguish PD from other disorders, including a standardized medical and neurological history, the Cognitive Abilities Screening Instrument (Teng et al. 1994), a scripted, standardized videotaped assessment of parkinsonism, a handwriting sample, medications, and the University of Pennsylvania brief smell identification test (Sensonics, Inc., Haddon Heights, NJ, USA) (Doty et al. 1996). Assessments by neurologists also included a standardized neurological examination, orthostatic hypotension assessment, the Unified Parkinson’s Disease Rating Scale (Fahn et al. 1987), and the Tremor Rating Scale (Fahn et al. 1993). Technician-assessed controls with signs suggesting parkinsonism were subsequently assessed by a neurologist.

Diagnosis was determined by agreement of two neurologists (C.M.T. and either G.W.R., C.M., A.C., or M.K.) after independent review of all available diagnostic information (medical records, in-person examination records, and videotaped examination) for all suspect cases and when PD was suspected in a control. Established criteria for PD and related disorders were used (Bain et al. 2000; Elble 2000; Gelb et al. 1999; Gilman et al. 1999; Hughes et al. 1992; Langston et al. 1992; Litvan et al. 1996; McKeith et al. 1996).

FAME was approved by institutional review boards for the Parkinson’s Institute, National Institutes of Health, Social and Scientific Systems, Inc. (Durham, NC, USA), the University of Iowa (Iowa field station), and Battelle, Inc. (North Carolina field station). All participants provided written informed consent.

### Exposure assessment

We used computer-assisted telephone interviews to obtain detailed information on pesticide use from 14 years of age onward for 31 selected pesticides [Supplemental Material, Table 1 (doi:10.1289/ehp.1002839)] as well as covariate information including lifelong smoking and family history of PD. For subjects who were unable to complete interviews, we used proxy informants. Although all controls were living and competent when enrolled, some later became unable to participate and a proxy informant was used. We considered only pesticide use occurring before a reference date (cases: age at PD diagnosis; controls: median age of PD diagnosis for cases within the corresponding age-, sex-, and state-specific stratum). For each pesticide, we determined ever use (used one or more times) and lifetime days of use (determined for each farm job by multiplying years of use by average days of use per year, and then summing across jobs). Self-reported pesticide use falling outside U.S. Environmental Protection Agency (EPA) approval dates was not included. For cigarette smoking, we classified individuals who smoked > 100 cigarettes before their reference date as exposed (Grant et al. 2004). Family history of PD was positive if the subject reported PD in any first-degree relative. We dichotomized educational level as ≤ 12 years and > 12 years.

### Statistical analyses

We compared participant characteristics using Fisher’s exact test or Pearson’s chi-square statistic for categorical data and the Wilcoxon rank-sum test for continuous data. For pesticides reported by ≥ 10 subjects, we evaluated associations between pesticide use and PD using unconditional logistic regression to derive odds ratios (ORs) and 95% confidence intervals (CIs) [Supplemental Material, Table 2 (doi:10.1289/ehp.1002839)]. To control potential confounding, we included reference age (tertiles 40–57, 58–65, 66–87 years), sex, state, and cigarette smoking (ever/never) in all models. We also examined whether adjusting for overall pesticide use or educational level changed point estimates (± 15%) for individual pesticides. To adjust for overall pesticide use, we categorized individuals who used pesticides on < 25 lifetime days as unexposed and others as exposed, using data reported at enrollment in the AHS (data from AHS data release version P1REL0506 and AHSREL0612; unpublished data).

We performed analyses for pesticide exposure groups classified by mechanism as complex I inhibitors or oxidative stressors, based on literature review (Degli Esposti 1998; Krieger 2001; Uversky et al. 2001) and information in public databases [Agency for Toxic Substances and Disease Registry (ATSDR) 2002; Extension Toxicology Network (EXTOXNET) 2002; National Center for Biotechnology Information 2002]. Because men were the primary users of pesticides, we repeated analyses of ever use and duration of use restricted to men. For each pesticide reported by > 30 men [Supplemental Material, Table 3 (doi:10.1289/ehp.1002839)], we assessed the effect of exposure duration by separately comparing less use (at or below the median number of cumulative days of use) or more use (use above the median) with never use of that pesticide.

We repeated primary analyses in subgroups defined by race/ethnicity (non-Hispanic whites, others), state, family history of PD, cigarette use, and respondent status (self-reported vs. proxy-reported pesticide data) and, in cases, PD duration. We performed analyses for rotenone and paraquat use truncated 5, 10, and 15 years before the index date to evaluate whether the association of PD with pesticide use was influenced by behavioral changes caused by undiagnosed disease. We also assessed the combination of paraquat plus any dithiocarbamate (ferbam, mancozeb, maneb, metam sodium, vegedex, zineb, ziram), because previous studies reported an association of PD with the combination of paraquat and the dithiocarbamate maneb (Costello et al. 2009; Thiruchelvam et al. 2000). We also repeated the primary analysis after including cases of atypical parkinsonism in our case group. For cases, we compared clinical features in persons who did or did not use paraquat or oxidative stressors as a group, or rotenone or complex I inhibitors as a group.

We used SAS version 9.1.3 (SAS Institute Inc., Cary, NC, USA) and SPSS version 12.0 (SPSS Inc., Chicago, IL, USA) for statistical analyses. We used α ≤ 0.05 as a criterion for statistical significance.

## Results

**Figure 1:**
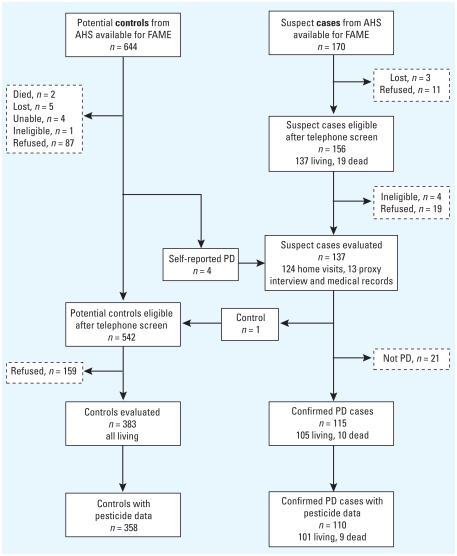
Screening process of cases and controls. We identified 170 suspect cases and 644 potential controls and screened 156 (92%) and 542 (84%) of these, respectively. We conducted study evaluations in 137 (88%) of eligible suspect cases (including four initially identified as potential controls who self-reported PD at screening) and 383 (71%) of eligible potential controls. Suspect cases were evaluated in home visits or from medical records, matched controls in home visits. Final diagnoses in suspect cases were PD (115), essential tremor/other tremor disorder (12), no neurologic diagnosis (5), dystonia (2), multiple system atrophy (2), and atypical parkinsonism not fulfilling any diagnostic criterion (1). The latter three cases were subsequently termed “atypical parkinsonism.”

We identified 170 suspect cases and 644 potential controls and screened 156 (92%) and 542 (84%) of these, respectively (Figure 1). We conducted study evaluations in 137 (88%) of eligible suspect cases (including 4 initially indentified as potential controls who self-reported PD at screening) and 383 (71%) of eligible potential controls. Final diagnoses in suspect cases were PD (115), essential tremor/other tremor disorder (12), no neurologic diagnosis (5), dystonia (2), multiple system atrophy (2), and atypical parkinsonism not fulfilling any diagnostic criterion (1), the latter three cases subsequently termed “atypical parkinsonism.”

The analyses reported here include 110 PD cases and 358 controls who provided complete information on pesticide use and application practices. Cases and controls were similar for most characteristics assessed (Table 2), although cases were more likely to have impaired smell recognition or to have a family history of PD, and were less likely to smoke.

To validate our approach to pesticide exposure assessment, we compared information on use of four pesticides collected at enrollment in the AHS with information collected for FAME. Despite an average lapse of 9 years, agreement was good [DDT (dichlorodiphenyltrichloroethane) 79%, 2,4-D (2,4-dichlorophenoxyacetic acid) 84%, paraquat 85%, rotenone 93%].

Ninety-eight percent of men and 44% of women had ever used pesticides. Information on 18 pesticides used by > 10 subjects is presented in Supplemental Material, Table 2 (doi:10.1289/ehp.1002839). Results from analyses in men were similar to those in men and women combined (Supplemental Material, Table 2). Information on pesticides linked to complex I inhibition or oxidative stress, regardless of the number of users, is presented in Table 3. Most of the pesticides in the oxidative stressor and mitochondrial inhibitor groups were used infrequently, limiting individual analyses; but in general, use of these chemicals was more common in cases. Use of paraquat (OR = 2.5; 95% CI, 1.4–4.7) or any of the group of oxidative stressors (OR = 2.0; 95% CI, 1.2–3.6) was associated with PD (Table 3). Similarly, use of rotenone (OR = 2.5; 95% CI, 1.3–4.7) or any of the group of complex I inhibitors (OR = 1.7; 95% CI, 1.0–2.8) was associated with PD (Table 3).

Associations between ever use of paraquat or rotenone and PD were similar when exposures were truncated at 5, 10, or 15 years before the diagnosis or reference date [paraquat: 5 years prior OR = 2.7 (95% CI, 1.4–4.9), 10 years OR = 2.9 (95% CI, 1.6–5.5), 15 years OR = 3.1 (95% CI, 1.6–5.8); rotenone: 5 years prior OR = 2.3 (95% CI, 1.2–4.4), 10 years OR = 2.4 (95% CI, 1.3–4.6), 15 years OR = 2.4 (95% CI, 1.3–4.6]. Exposures to rotenone and paraquat were not correlated (*r* = 0.004), and ORs from a model that included both pesticides (paraquat OR = 2.6; 95% CI, 1.4–4.9; rotenone OR = 2.9; 95% CI, 1.5–5.5) were comparable with those from models without mutual adjustment (Table 3). For men who used paraquat plus any dithiocarbamate (7 cases, 16 controls), the OR compared with use of neither was 2.2 (95% CI, 0.79–5.9). Compared with never users, associations with PD were generally stronger among those who had used pesticides for more than the median number of lifetime days than among those who had used pesticides for fewer than the median number of days [Supplemental Material, Table 3 (doi:10.1289/ehp.1002839)].

Results from analyses stratified by race/ethnicity, cigarette use, state, or duration of disease (in cases) were not appreciably different between subgroups (data not shown). In an analysis restricted to 18 cases and 22 controls (28 men and 12 women) with a first-degree relative with PD, PD was not associated with ever exposure to rotenone (OR = 0.98) or paraquat (OR = 1.1). Adjustment for education or overall pesticide use did not appreciably alter effect estimates, nor did exclusion of 28 cases and 6 controls with proxy interviews or inclusion of 3 atypical parkinsonism cases.

We compared clinical features of cases who did or did not use paraquat or any oxidative stressor, or rotenone or any complex I inhibitor (Table 4). PD was diagnosed at a younger age among those who used oxidative stressors (mean age = 59 vs. 64 years, *p* = 0.02). Although postural reflex impairment appeared to be less frequent in those using either paraquat or rotenone, these differences were not statistically significant. Clinical features did not otherwise differ between groups. We were unable to determine whether exposure to both paraquat and rotenone was associated with unique clinical features because only five cases reported use of both pesticides (data not shown).

## Discussion

The pathogenesis of PD is thought to involve several critical abnormalities, each of which can be the result of genetic or environmental factors. Chief among these abnormalities are dysfunction of the mitochondrial respiratory chain, particularly complex I, and the production of reactive oxygen species (Henchcliffe and Beal 2008). To our knowledge, we have performed the first analysis of pesticides classified by presumed mechanism, rather than by functional categories (e.g., herbicides) or chemical class (e.g., organochlorines). We found significant associations of PD with use of groups of pesticides classified as complex I inhibitors or as oxidative stressors, providing support in humans for findings from decades of experimental work. In particular, PD was strongly associated with rotenone and paraquat, two individual pesticides used extensively to model PD in the laboratory.

This study provides strong evidence of an association between rotenone use and PD in humans. PD developed 2.5 times as often in those who reported use of rotenone compared with nonusers, and an association of similar magnitude was observed even when exposure was truncated up to 15 years before PD diagnosis. In our prior analysis of self-reported PD in the AHS, information on rotenone was available for a small subgroup, and nonsignificant association with PD (OR = 1.7; 95% CI, 0.6–4.7) was observed (Kamel et al. 2007), whereas in a multicenter, clinic-based case–control study distinct from the AHS, only two individuals were exposed and no association was observed (Tanner et al. 2009). In the only other report of rotenone-like compounds and PD, use of organic pesticides such as rotenone in the previous year was determined for PD clinic attendees, who reported current use more often than did cases with other neurologic diseases (Dhillon et al. 2008). This information cannot be used to assess etiology, because the study evaluated associations with rotenone use that occurred after PD had been diagnosed. In contrast, in the present population-based study, we evaluated rotenone use before PD diagnosis in cases and during a comparable time period in neurologically healthy controls.

Rotenone is a plausible cause of PD because of its mechanism of action. Like 1-methyl-4-phenyl-1,2,3,6-tetrahydropyridine (MPTP), a toxicant known to cause parkinsonism in humans, rotenone directly inhibits mitochondrial complex I (Langston et al. 1983; Sherer et al. 2007). In experimental models, both MPTP and rotenone cause selective injury of dopaminergic neurons in the substantia nigra, a key pathological feature of PD (Greenamyre et al. 1999; Langston et al. 1984). Because rotenone is believed to have a relatively short environmental half-life and limited bioavailability, a relationship to human disease has been questioned (Hatcher et al. 2008; Li et al. 2005). However, recent work in rodent models indicated that a temporally limited exposure to rotenone later caused progressive functional and pathologic changes in the enteric nervous system of rodents, mimicking changes found in human PD; as in PD, these enteric nervous system changes preceded central nervous system pathology (Abbott et al. 2001; Braak et al. 2006; Drolet et al. 2009; Greene et al. 2009; Pan-Montojo et al. 2010). Chronic rotenone exposure in the laboratory has been reported to have additional effects associated with PD pathogenesis, including ones similar to changes observed in monogenic forms of PD (Henchcliffe and Beal 2008). Rotenone toxicity, therefore, provides a conceptual bridge, suggesting shared mechanisms for both sporadic and inherited forms of PD.

Although we report here findings for agricultural use of rotenone, the ubiquitous use of rotenone in both work and home settings that occurred until recently suggests that many people may have been exposed. Humans have used rotenone-containing plants as pesticides for centuries (Cabras et al. 2002). Because rotenone is plant derived, it has been considered an organic pesticide and was commonly used as a household insecticide in home gardening and agriculture, and to kill fish. For example, the California Department of Pesticide Regulation (2007) reported that almost 15,000 pounds of rotenone were used in 2007, not including home use. Rotenone was withdrawn from use in the European Union in 2007 (Schapira 2010), after which time most uses were voluntarily cancelled in the United States (U.S. EPA 2007). Other agents associated with mitochondrial complex I inhibition remain in common use. For example, permethrin is used in nonagricultural settings as an insect repellant, including use of permethrin-impregnated fabric for military uniforms and recreational clothing (Armed Forces Pest Management Board 2010).

Our study also extends prior research on paraquat. Experimentally, paraquat produces subcellular changes associated with PD, including increased production of reactive oxygen species, alpha-synuclein aggregation, and selective nigral injury (Dinis-Oliveira et al. 2006; Kuter et al. 2010; McCormack et al. 2002). Previously, we found an association between paraquat use and PD in prevalent but not incident self-reported cases in the AHS (Kamel et al. 2007) and a nonsignificant association between PD and occupational paraquat use in a multicenter case–control study (Tanner et al. 2009). Cumulative use was not assessed in either study. Only a few other studies have assessed associations between PD and paraquat use (Table 1). A study of 120 cases and 240 controls conducted in Taiwan (Liou et al. 1997) reported an OR of 3.22 (95% CI, 2.41–4.31) for PD in paraquat users compared with nonusers. Cumulative exposure was associated with greater risk, but paraquat use in the Taiwanese study was highly correlated with use of other herbicides. Although the inconsistency of findings in human populations has been used as a basis for suggesting that paraquat is not associated with PD (Li et al. 2005; Miller 2007), an alternative explanation is that few studies have had adequate size and sufficiently detailed exposure information to allow the association to be observed. Our findings, considered together with earlier results, suggest that paraquat use plays a role in human PD. Because paraquat remains one of the most widely used herbicides worldwide (Frabotta 2009), this finding potentially has great public health significance.

Parkinsonism in humans due to high-dose exposure to toxicants such as carbon monoxide or manganese has characteristic clinical features including less prominent tremor, more prominent postural instability, symmetric distribution of signs, and poor response to dopaminergic therapy (Tanner 1992). We did not observe such features in our cases. Cases who did or did not use rotenone, paraquat, or groups of pesticides with similar mechanisms were generally similar, suggesting that PD associated with these agents is clinically typical and indirectly supporting a role for pesticide exposure in the etiology of typical PD. We did note an earlier age at diagnosis in users of oxidative stressors and a suggestion of this in paraquat users specifically. Early age at onset is also a characteristic of genetic parkinsonism in which oxidative stress is a presumed pathophysiologic mechanism (*alpha-synuclein, PINK-1, DJ-1*, and *PARKIN* mutations) (Henchcliffe and Beal 2008; Klein et al. 2009).

In FAME, pesticide exposure was not associated with PD in individuals with a family history, although numbers were small. Interestingly, Hancock et al. (2008) similarly found pesticide exposure to be associated with PD risk only in those without an affected first-degree relative.

Our study has some limitations. First, because most participants were exposed to many pesticides, we cannot confidently exclude effects of agents other than those studied or rule out the possibility that our results are attributable to combined exposures. However, the associations that we observed remained after adjustment for overall pesticide use, and estimated effects of rotenone and paraquat were comparable after mutual adjustment. Future investigations of combinations of pesticides and of other mechanistic groups will be important. Second, we could not use laboratory measures of pesticides or their metabolites to estimate exposure. Such measures are not available for many of the pesticides we studied, and when available, they are poor predictors of past or long-term use. Thus, although we recognize that retrospective exposure assessment has limitations, it is often the best approach for studying lifelong exposure in an adult population in connection with a rare disease. Third, we included prevalent cases already diagnosed but still living at enrollment in the AHS; therefore, survivor bias is possible. However, our results were similar when only those with shorter disease duration were analyzed. Additionally, we were able to investigate only persons willing to participate. Thus, PD cases or controls in this study may not have been fully representative of the entire population. However, participation was good, partially allaying this concern. Finally, we selected pesticides presumed to act through specific toxic mechanisms, but for most pesticides there is very little information regarding toxic effects in humans, as most studies are directed toward effects on plant or animal pests. It is likely that we have misclassified some pesticides with regard to mechanism. However, the likely effect of any misclassification would be to attenuate an association with pesticides grouped according to a common mechanism.

Strengths include the size of the study; the focus on an agricultural cohort with many exposed individuals and wide variability in exposure; the quality of diagnosis, which was based on in-person assessment and agreement of movement disorders experts; and the completeness and reliability of the pesticide exposure information. An additional strength is the nested case–control design with an internal control group who had similar exposure opportunities as the cases and similar demographic and lifestyle characteristics, reducing the likelihood of bias or confounding. Use of pesticides in general was ubiquitous, of course, in applicators and relatively common among their spouses, and all participants may have had additional passive pesticide exposure. However, these features would all be likely to lower the chance of identifying any effect.

Our study helps connect the dots between basic research and human populations. Rotenone and paraquat have been linked experimentally to pathophysiological mechanisms implicated in human PD. Groups of pesticides linked to the mechanisms of mitochondrial dysfunction or oxidative stress were also associated with PD in our study, thus extending experimental work to provide strong evidence that these mechanisms play a role in PD in humans. Importantly, the potential for exposure to many of these pesticides, including rotenone and paraquat, extends well beyond the occupational setting. Many persons with nonoccupational pesticide exposures may be unaware of the presence of pesticides in their environments (Centers for Disease Control and Prevention 2009). The potential for exposure to other toxicants with similar mechanisms is even greater. To continue the interplay between human and experimental studies, future mechanistic studies of these pesticides should model exposure conditions similar to those occurring in humans, including chronic low-dose exposure, exposure to multiple agents, and assessment of gene–exposure interactions. Such work could provide new insights into the pathogenesis and ultimately the prevention of PD.

## Figures and Tables

**Table 1:** Relationship of PD to paraquat or rotenone exposure in human populations.

Study	Design	Method of assessing pesticide use	No. enrolled (cases, controls)	Finding	OR (95% CI) or *p*-value	Exposed *n*
Paraquat						
Sanchez-Ramos et al. 1987	Case report	Medical history	1 case	Symptoms “comparable to PD” in a 32-year-old farmer after 15 years of paraquat use	NA	1 case

Hertzman et al. 1990	Case–control	Specific question re: paraquat use	57 cases	Association with PD	*p* = 0.0*^a^	4 cases
			122 controls			0 controls

Semchuk et al. 1992	Case–control	General questions re: pesticide use	130 cases	One case with early-onset PD (< age 40 years) reported using paraquat (ages 26–31 years)	NA	1 case
			260 cases			

Hertzman et al. 1994	Case–control	Specific question re: paraquat use	127 cases		1.11 (0.32–3.87)	6 cases
			245 controls	No association with PD		5 controls
					1.25 (0.34–4.63)	6 cases
						4 controls

Liou et al. 1997	Case–control	Open-ended question re: pesticide use	120 cases	No association with PD	3.22 (2.41–4.31)	31 cases
			240 controls			22 controls

Firestone et al. 2005	Case–control	Checklist^b^	250 cases	Association with PD	1.67 (0.22–12.76)	2 cases
			388 controls			2 controls

Kamel et al. 2007	Case–control	Specific question re: paraquat use	83 prevalent cases	Association with PD in prevalent cases	1.8 (1.0–3.4)	14 prevalent
			79,557 controls			11,266 controls
			78 incident cases	No association with PD in incident cases	1.0 (0.5–1.9)	11 incident
			55,931 controls			7,382 controls

Tanner et al. 2009	Case–control	Specific question re: occupational paraquat use	519 cases	Association with PD	2.80 (0.81–9.72)	9 cases
			511 controls			4 controls

Firestone et al. 2010	Case–control	Checklist^c^	404 incident cases	Added subjects to 2005 interim population	0.9 (0.14–5.43)	2 cases
			526 controls	No association with PD in reanalysis		3 controls

Rotenone						
Kamel et al. 2007	Case–control	Specific question re: rotenone use in a supplementary questionnaire	83 prevalent cases	Association of PD with ever use	1.7 (0.6–4.7)	4 prevalent
			79,557 controls			671 controls
			78 incident cases	Could not determine		1 incident
			55,931 controls			565 controls

Tanner et al. 2009	Case–control	Specific question re: occupational rotenone use	519 cases	No association with PD	0.82 (0.05–13.34)	1 case
			511 controls			1 control

Dhillon et al. 2008	Case–control	General question re: “organic pesticides”	100 cases	Greater use of “organic pesticides such as rotenone” in PD patients	10.0 (2.9–34.3)	27 cases
			84 controls			3 controls

NA, not analyzed.

a Four PD patients and no controls reported paraquat contact; OR could not be calculated;

b Occupational pesticide exposures were identified from a checklist of common chemical agents and home-based pesticide exposures from a checklist of commercial brand name products.

c Subjects reported exposures to various industrial toxicants identified from a checklist.

**Table 2:** Characteristics of subjects.

Characteristic	Cases	Controls
*n*	110	358
Age at FAME enrollment [years (mean ± SD)]	70 ± 8	69 ± 8
Men [*n* (%)]	80 (73)	265 (74)
Residence in Iowa (vs. North Carolina) [*n* (%)]	79 (72)	262 (73)
Non-Hispanic white (vs. other) [*n* (%)]	107 (97)	350 (98)
Pesticide applicator (vs. spouse) [*n* (%)]	80 (73)	267 (75)
Education > high school [*n* (%)]	49 (45)	178 (50)
PD in first-degree relative [*n* (%)]	18 (16)	22 (6)*
Smoked at least 100 cigarettes [*n* (%)]	31 (28)	141 (39)**
BSIT (mean score ± SD)	5.5 ± 2.8	8.8 ± 2.3#
CASI (mean score ± SD)	89.2 ± 8.5	93.1 ± 5.0#
Clinical features among cases^a^		
Age at PD diagnosis [years (mean ± SD)]	61 ± 9	—
PD duration at FAME enrollment [years (median ± SD)]	7 ± 6	—
Resting tremor [*n* affected/*n* with data (%)]	100/108 (93)	—
Bradykinesia [*n* affected/*n* with data (%)]	105/110 (95)	—
Rigidity [*n* affected/*n* with data (%)]	106/107 (99)	—
Postural reflex impairment [*n* affected/*n* with data (%)]	61/92 (66)	—
Asymmetric onset [*n* affected/*n* with data (%)]	102/104 (98)	—
Response to dopaminergic therapy (if prescribed) [*n* affected/*n* with data (%)]	95/98 (97)	—

Abbreviations: BSIT, Brief Smell Identification Test; CASI, Cognitive Abilities Screening Instrument.

a Percentages for clinical features are based on numbers of cases with features divided by the total number of cases with available data; cases with missing data for a feature are excluded.

* *p* < 0.005 (Fisher’s exact test).

** *p* < 0.05 (Fisher’s exact test).

# *p* < 0.001 (Wilcoxon rank-sum test).

**Table 3:** Association of PD with ever use of pesticides before diagnosis or reference date by mechanism.

Pesticide	Cases (*n* = 110) [*n* (%)]	Controls (*n* = 358) [*n* (%)]	OR (95% CI)	*p-*Value
Oxidative stressors				
Paraquat	23 (24)	49 (14)	2.5 (1.4–4.7)	0.004
Permethrin	16 (16)	41 (12)	1.5 (0.77–2.9)	0.244
Carbon disulfide	2 (2)	3 (1)	2.6 (0.41–16)	0.313
Chloranil	1 (1)	3 (1)	1.6 (0.16–16)	0.706
Cyhalothrin	1 (1)	1 (0)	3.8 (0.22–64)	0.359
Dichlone	3 (3)	8 (2)	1.6 (0.40–6.2)	0.517
Mercury compounds	2 (2)	5 (1)	1.4 (0.26–7.5)	0.692
Pybuthrin	0 (0)	6 (2)	NA	
Any oxidative stressor	35 (40)	93 (28)	2.0 (1.2–3.6)	0.012

Mitochondrial complex I inhibitors				
Benomyl	7 (7)	15 (4)	1.9 (0.70–5.0)	0.207
Carbendazim	1 (1)	2 (1)	2.2 (0.19–25)	0.529
Cyhalothrin	1 (1)	1 (0)	3.8 (0.22–64)	0.359
Permethrin	16 (16)	41 (12)	1.5 (0.77–2.9)	0.244
Pyridaben	0 (0)	1 (0)	NA	
Rotenone	19 (19)	32 (9)	2.5 (1.3–4.7)	0.005
Thiabendazole	3 (3)	12 (3)	0.8 (0.23–3.1)	0.778
Any complex I inhibitor	36 (38)	92 (27)	1.7 (1.0–2.8)	0.041

NA, not available. Analyses used logistic regression adjusted for reference age tertile, sex, state, and cigarette smoking.

**Table 4:** Clinical features of PD cases by use of paraquat or rotenone or mechanistic group.

	Paraquat		Any oxidative stressor		Rotenone		Any complex I inhibitor	
Characteristic	Exposed (*n =* 23)	Not exposed (*n =* 74)	Exposed (*n =* 35)	Not exposed (*n =* 53)	Exposed (*n =* 19)	Not exposed (*n =* 82)	Exposed (*n =* 36)	Not exposed (*n =* 59)
Age at PD diagnosis [years (mean ± SD)]	59 ± 8	62 ± 9	59 ± 8	64 ± 9*	65 ± 11	61 ± 8	61 ± 11	62 ± 8
PD duration [years (mean ± SD)]	8.6 ± 6	8.4 ± 6	8.2 ± 6	7.6 ± 5	7.7 ± 7	8.7 ± 5	8.2 ± 6	8.5 ± 5
Clinical features [*n* with feature/*n* with data available (%)]								
Resting tremor	21/22 (95)	66/73 (90)	31/34 (91)	49/53 (92)	17/18 (94)	75/81 (93)	32/35 (91)	54/58 (93)
Bradykinesia	22/23 (96)	70/74 (95)	33/35 (94)	50/53 (94)	17/19 (89)	79/82 (96)	34/36 (94)	56/59 (95)
Rigidity	23/23 (100)	72/73 (99)	33/34 (97)	50/53 (94)	18/19 (95)	80/80 (100)	34/35 (97)	58/58 (100)
Postural reflex impairment	10/21 (48)	43/62 (69)	18/31 (58)	29/43 (67)	7/15 (47)	48/69 (70)	16/28 (57)	37/52 (71)
Asymmetric onset	23/23 (100)	69/71 (97)	35/35 (100)	49/51 (96)	18/18 (100)	77/79 (97)	35/35 (100)	54/56 (96)
Response to dopaminergic therapy (if prescribed)	21/21 (100)	63/65 (97)	30/30 (100)	46/48 (96)	14/15 (93)	73/74 (99)	30/31 (97)	53/54 (98)

* Difference between exposed versus not exposed *p* = 0.02.

## References

[b1-ehp-119-866] Abbott RD, Petrovitch H, White LR, Masaki KH, Tanner CM, Curb JD (2001). Frequency of bowel movements and the future risk of Parkinson’s disease. Neurology.

[b2-ehp-119-866] Alavanja MC, Sandler DP, McMaster SB, Zahm SH, McDonnell CJ, Lynch CF (1996). The Agricultural Health Study. Environ Health Perspect.

[b3-ehp-119-866] Armed Forces Pest Management Board (2010). Permethrin – Impregnated Clothing.

[b4-ehp-119-866] ATSDR (Agency for Toxic Substances and Disease Registry) (2002). Home page.

[b5-ehp-119-866] Bain P, Brin M, Deuschl G, Elble R, Jankovic J, Findley L (2000). Criteria for the diagnosis of essential tremor. Neurology.

[b6-ehp-119-866] Betarbet R, Sherer TB, MacKenzie G, Garcia-Osuna M, Panov AV, Greenamyre JT (2000). Chronic systemic pesticide exposure reproduces features of Parkinson’s disease. Nat Neurosci.

[b7-ehp-119-866] Blair A, Tarone R, Sandler D, Lynch CF, Rowland A, Wintersteen W (2002). Reliability of reporting on life-style and agricultural factors by a sample of participants in the Agricultural Health Study from Iowa. Epidemiology.

[b8-ehp-119-866] Braak H, de Vos RA, Bohl J, Del Tredici K (2006). Gastric alpha-synuclein immunoreactive inclusions in Meissner’s and Auerbach’s plexuses in cases staged for Parkinson’s disease-related brain pathology. Neurosci Lett.

[b9-ehp-119-866] Cabras P, Caboni P, Cabras M, Angioni A, Russo M (2002). Rotenone residues on olives and in olive oil. J Agric Food Chem.

[b10-ehp-119-866] California Department of Pesticide Regulation (2007). Pesticide Use Reporting (PUR).

[b11-ehp-119-866] Centers for Disease Control and Prevention (2009). Fourth National Report on Human Exposure to Environmental Chemicals.

[b12-ehp-119-866] Costello S, Cockburn M, Bronstein J, Zhang X, Ritz B (2009). Parkinson’s disease and residential exposure to maneb and paraquat from agricultural applications in the central valley of California. Am J Epidemiol.

[b13-ehp-119-866] Degli Esposti M (1998). Inhibitors of NADH-ubiquinone reductase: an overview. Biochim Biophys Acta.

[b14-ehp-119-866] Dhillon AS, Tarbutton GL, Levin JL, Plotkin GM, Lowry LK, Nalbone JT (2008). Pesticide/environmental exposures and Parkinson’s disease in East Texas. J Agromedicine.

[b15-ehp-119-866] Di Monte DA, Lavasani M, Manning-Bog AB (2002). Environmental factors in Parkinson’s disease. Neurotoxicology.

[b16-ehp-119-866] Dinis-Oliveira RJ, Remiao F, Carmo H, Duarte JA, Navarro AS, Bastos ML (2006). Paraquat exposure as an etiological factor of Parkinson’s disease. Neurotoxicology.

[b17-ehp-119-866] Doty RL, Marcus A, Lee WW (1996). Development of the 12-item Cross-Cultural Smell Identification Test (CC-SIT). Laryngoscope.

[b18-ehp-119-866] Drolet RE, Cannon JR, Montero L, Greenamyre JT (2009). Chronic rotenone exposure reproduces Parkinson’s disease gastrointestinal neuropathology. Neurobiol Dis.

[b19-ehp-119-866] Elble RJ (2000). Diagnostic criteria for essential tremor and differential diagnosis. Neurology.

[b20-ehp-119-866] EXTOXNET (Extension Toxicology Network) (2002). Home page.

[b21-ehp-119-866] Fahn S, Elton RL, Fahn S, Marsden CD, Calne DB, Goldstein M, Members of the UPDRS Development Committee (1987). Unified Parkinson’s disease rating scale. Recent Developments in Parkinson’s Disease.

[b22-ehp-119-866] Fahn S, Tolosa E, Marin C, Jankovic J, Tolosa E (1993). Clinical rating scale for tremor. Parkinson’s Disease and Movement Disorders.

[b23-ehp-119-866] Firestone JA, Lundin JI, Powers KM, Smith-Weller T, Franklin GM, Swanson PD (2010). Occupational factors and risk of Parkinson’s disease: a population-based case-control study. Am J Ind Med.

[b24-ehp-119-866] Firestone JA, Smith-Weller T, Franklin G, Swanson P, Longstreth WT, Checkoway H (2005). Pesticides and risk of Parkinson disease: a population-based case-control study. Arch Neurol.

[b25-ehp-119-866] Frabotta D (2009). Paraquat: Herbicide Helps No-till Operations.

[b26-ehp-119-866] Gelb DJ, Oliver E, Gilman S (1999). Diagnostic criteria for Parkinson disease. Arch Neurol.

[b27-ehp-119-866] Gilman S, Low PA, Quinn N, Albanese A, Ben-Shlomo Y, Fowler CJ (1999). Consensus statement on the diagnosis of multiple system atrophy. J Neurol Sci.

[b28-ehp-119-866] Grant BF, Hasin DS, Chou SP, Stinson FS, Dawson DA (2004). Nicotine dependence and psychiatric disorders in the United States: results from the national epidemiologic survey on alcohol and related conditions. Arch Gen Psychiatry.

[b29-ehp-119-866] Greenamyre JT, MacKenzie G, Peng TI, Stephans SE (1999). Mitochondrial dysfunction in Parkinson’s disease. Biochem Soc Symp.

[b30-ehp-119-866] Greene JG, Noorian AR, Srinivasan S (2009). Delayed gastric emptying and enteric nervous system dysfunction in the rotenone model of Parkinson’s disease. Exp Neurol.

[b31-ehp-119-866] Hancock DB, Martin ER, Mayhew GM, Stajich JM, Jewett R, Stacy MA (2008). Pesticide exposure and risk of Parkinson’s disease: a family-based case-control study. BMC Neurol.

[b32-ehp-119-866] Hatcher JM, Pennell KD, Miller GW (2008). Parkinson’s disease and pesticides: a toxicological perspective. Trends Pharmacol Sci.

[b33-ehp-119-866] Hertzman C, Wiens M, Bowering D, Snow B, Calne D (1990). Parkinson’s disease: a case-control study of occupational and environmental risk factors. Am J Ind Med.

[b34-ehp-119-866] Hertzman C, Wiens M, Snow B, Kelly S, Calne D (1994). A case-control study of Parkinson’s disease in a horticultural region of British Columbia. Mov Disord.

[b35-ehp-119-866] Henchcliffe C, Beal MF (2008). Mitochondrial biology and oxidative stress in Parkinson disease pathogenesis. Nat Clin Pract Neurol.

[b36-ehp-119-866] Hoppin JA, Yucel F, Dosemeci M, Sandler DP (2002). Accuracy of self-reported pesticide use duration information from licensed pesticide applicators in the Agricultural Health Study. J Expo Anal Environ Epidemiol.

[b37-ehp-119-866] Hughes AJ, Daniel SE, Kilford L, Lees AJ (1992). Accuracy of clinical diagnosis of idiopathic Parkinson’s disease: a clinico-pathological study of 100 cases. J Neurol Neurosurg Psychiatry.

[b38-ehp-119-866] Kamel F, Tanner C, Umbach D, Hoppin J, Alavanja M, Blair A (2007). Pesticide exposure and self-reported Parkinson’s disease in the agricultural health study. Am J Epidemiol.

[b39-ehp-119-866] Klein C, Schneider SA, Lang AE (2009). Hereditary parkinsonism: Parkinson disease look-alikes—an algorithm for clinicians to “PARK” genes and beyond. Mov Disord.

[b40-ehp-119-866] Krieger R (2001). Handbook of Pesticide Toxicology.

[b41-ehp-119-866] Kuter K, Nowak P, Golembiowska K, Ossowska K (2010). Increased reactive oxygen species production in the brain after repeated low-dose pesticide paraquat exposure in rats. A comparison with peripheral tissues. Neurochem Res.

[b42-ehp-119-866] Langston JW, Ballard P, Tetrud JW, Irwin I (1983). Chronic Parkinsonism in humans due to a product of meperidine-analog synthesis. Science.

[b43-ehp-119-866] Langston JW, Forno LS, Rebert CS, Irwin I (1984). Selective nigral toxicity after systemic administration of 1-methyl-4-phenyl-1,2,5,6-tetrahydropyrine (MPTP) in the squirrel monkey. Brain Res.

[b44-ehp-119-866] Langston JW, Widner H, Goetz CG, Brooks DJ, Fahn S, Freeman TB (1992). Core assessment program for intracerebral transplantations (CAPIT). Mov Disord.

[b45-ehp-119-866] Li AA, Mink PJ, McIntosh LJ, Teta MJ, Finley B (2005). Evaluation of epidemiologic and animal data associating pesticides with Parkinson’s disease. J Occup Environ Med.

[b46-ehp-119-866] Liou HH, Tsai MC, Chen CJ, Jeng JS, Chang YC, Chen SY (1997). Environmental risk factors and Parkinson’s disease: a case-control study in Taiwan. Neurology.

[b47-ehp-119-866] Litvan I, Agid Y, Calne D, Campbell G, Dubois B, Duvoisin RC (1996). Clinical research criteria for the diagnosis of progressive supranuclear palsy (Steele-Richardson-Olszewski syndrome): report of the NINDS-SPSP international workshop. Neurology.

[b48-ehp-119-866] McCormack AL, Thiruchelvam M, Manning-Bog AB, Thiffault C, Langston JW, Cory-Slechta DA (2002). Environmental risk factors and Parkinson’s disease: selective degeneration of nigral dopaminergic neurons caused by the herbicide paraquat. Neurobiol Dis.

[b49-ehp-119-866] McKeith IG, Galasko D, Kosaka K, Perry EK, Dickson DW, Hansen LA (1996). Consensus guidelines for the clinical and pathologic diagnosis of dementia with Lewy bodies (DLB): report of the consortium on DLB international workshop. Neurology.

[b50-ehp-119-866] Miller GW (2007). Paraquat: the red herring of Parkinson’s disease research. Toxicol Sci.

[b51-ehp-119-866] National Center for Biotechnology Information (2002). PubChem.

[b52-ehp-119-866] Pan-Montojo F, Anichtchik O, Dening Y, Knels L, Pursche S, Jung R (2010). Progression of Parkinson’s disease pathology is reproduced by intragastric administration of rotenone in mice. PLoS One.

[b53-ehp-119-866] Sanchez-Ramos JR, Hefti F, Weiner WJ (1987). Paraquat and Parkinson’s disease. Neurology.

[b54-ehp-119-866] Schapira AH (2010). Complex I: inhibitors, inhibition and neurodegeneration. Exp Neurol.

[b55-ehp-119-866] Semchuk KM, Love EJ, Lee RG (1992). Parkinson’s disease and exposure to agricultural work and pesticide chemicals. Neurology.

[b56-ehp-119-866] Sherer TB, Richardson JR, Testa CM, Seo BB, Panov AV, Yagi T (2007). Mechanism of toxicity of pesticides acting at complex I: relevance to environmental etiologies of Parkinson’s disease. J Neurochem.

[b57-ehp-119-866] Tanner CM (1992). Occupational and environmental causes of parkinsonism. Occup Med.

[b58-ehp-119-866] Tanner CM, Ross GW, Jewell SA, Hauser RA, Jankovic J, Factor SA (2009). Occupation and risk of parkinsonism: a multicenter case-control study. Arch Neurol.

[b59-ehp-119-866] Teng EL, Hasegawa K, Homma A, Imai Y, Larson E, Graves A (1994). The Cognitive Abilities Screening Instrument (CASI): a practical test for cross-cultural epidemiological studies of dementia. Int Psychogeriatr.

[b60-ehp-119-866] Thiruchelvam M, Brockel BJ, Richfield EK, Baggs RB, Cory-Slechta DA (2000). Potentiated and preferential effects of combined paraquat and maneb on nigrostriatal dopamine systems: environmental risk factors for Parkinson’s disease?. Brain Res.

[b61-ehp-119-866] Tolosa E, Wenning G, Poewe W (2006). The diagnosis of Parkinson’s disease. Lancet Neurol.

[b62-ehp-119-866] U.S. EPA (U.S. Environmental Protection Agency) (2007). Reregistration Eligibility Decision for Rotenone. March 2007 EPA 738-R-07-005.

[b63-ehp-119-866] Uversky VN, Li J, Fink AL (2001). Pesticides directly accelerate the rate of alpha-synuclein fibril formation: a possible factor in Parkinson’s disease. FEBS Lett.

